# Carbon Emission and Biodiversity of Arctic Soil Microbial Communities of the Novaya Zemlya and Franz Josef Land Archipelagos

**DOI:** 10.3390/microorganisms11020482

**Published:** 2023-02-15

**Authors:** Zorigto Namsaraev, Anna Bobrik, Aleksandra Kozlova, Anastasia Krylova, Anastasia Rudenko, Anastasia Mitina, Aleksandr Saburov, Maksim Patrushev, Olga Karnachuk, Stepan Toshchakov

**Affiliations:** 1 Kurchatov Centre for Genome Research, NRC “Kurchatov Institute”, 123182 Moscow, Russia; 2 NBIC Department, Moscow Institute of Physics and Technology, 141701 Dolgoprudny, Russia; 3 Soil Science Faculty, Moscow State University, 119991 Moscow, Russia; 4 Northern (Arctic) Federal University, 163002 Arkhangelsk, Russia; 5 Department of Plant Physiology, Biotechnology, and Bioinformatics, Tomsk State University, 634050 Tomsk, Russia

**Keywords:** biodiversity-ecosystem functioning, arctic soil, arctic microbial diversity, CO_2_ emission, Novaya Zemlya, Franz Josef Land, Barents Sea, soil carbon

## Abstract

Cryogenic soils are the most important terrestrial carbon reservoir on the planet. However, the relationship between soil microbial diversity and CO_2_ emission by cryogenic soils is poorly studied. This is especially important in the context of rising temperatures in the high Arctic which can lead to the activation of microbial processes in soils and an increase in carbon input from cryogenic soils into the atmosphere. Here, using high-throughput sequencing of 16S rRNA gene amplicons, we analyzed microbial community composition and diversity metrics in relation to soil carbon dioxide emission, water-extractable organic carbon and microbial biomass carbon in the soils of the Barents Sea archipelagos, Novaya Zemlya and Franz Josef Land. It was found that the highest diversity and CO_2_ emission were observed on the Hooker and Heiss Islands of the Franz Josef Land archipelago, while the diversity and CO_2_ emission levels were lower on Novaya Zemlya. Soil moisture and temperature were the main parameters influencing the composition of soil microbial communities on both archipelagos. The data obtained show that CO_2_ emission levels and community diversity on the studied islands are influenced mostly by a number of local factors, such as soil moisture, microclimatic conditions, different patterns of vegetation and fecal input from animals such as reindeer.

## 1. Introduction

Soil microorganisms are critical to the function of soil ecosystems, including methanogenesis, soil respiration, nitrogen cycling, and other processes [1]. However, the relationship between microbial diversity and soil ecosystem function is poorly understood. According to recent estimates, only 0.3% of soil sampling locations have received information on both biodiversity and soil ecosystem functioning [2]. Information on biodiversity ecosystem functioning (BEF) for ecosystems with low productivity is particularly scarce [3]. 

Cryogenic soils occupy 15% of all soils while storing 50% of the total soil organic carbon, and thus they are the most important terrestrial carbon reservoirs on the planet [4]. For hundreds of years, cryogenic soils have accumulated organic carbon and nitrogen under the conditions of a short growing season, low temperatures, constantly high humidity, and permafrost [5]. McGuire and coauthors estimate that the pool of organic C in cryogenic soils (1400–1850 × 10^15^ g) exceeds the pools of organic C in the atmosphere (750 × 10^15^ g), ocean (1000 × 10^15^ g) and terrestrial vegetation (560 × 10^15^ g). It is comparable to the organic C pool (1500 × 10^15^ g) in non-cryogenic soils [6]. Organic matter preserved in permafrost plays an important role in the global carbon cycle, as its decomposition by microorganisms during climate warming may lead to the emission of significant amounts of greenhouse gases into the atmosphere [7,8,9]. The additional emission of carbon into the atmosphere in the form of CO_2_ and CH_4_ will further increase the greenhouse effect via the feedback principle, and its amount may become comparable with the annual anthropogenic increase in these gases in the atmosphere. This may lead to a conversion of northern ecosystems from sinks of organic carbon to their sources. CO_2_ emission from cryogenic soils is an integral indicator of their biological activity [10]. The number of studies on greenhouse gas emission and the organic carbon contentof soils of Arctic ecosystems is very limited. There is also very limited information on the relationship between microbial diversity and the functioning of Arctic ecosystems [11,12,13].

An increase in air temperature has been noted in many regions of the Arctic and subarctic zone [14,15]. Over the last 30 years, an average air temperature increase in the Arctic region of 0.09 °C/10 years has been found, which is two times higher than in other regions of the northern hemisphere [14]. The northern part of the Barents Sea is one of the most rapidly warming places on Earth, with the strongest increase in the temperature of the lower atmosphere, the greatest loss of winter sea ice and a rapidly warming ocean [16,17]. These processes have intensified particularly since the mid-2000s, increasing the likelihood that this region may experience a transition from a cold Arctic to a warmer Atlantic climate regime in the coming years, with implications for Barents Sea archipelago ecosystems. 

The relationship between biodiversity and soil ecosystem functioning in the Barents Sea archipelagos is poorly understood. More information is available on the soil communities of the Spitsbergen archipelago [18,19,20,21], but the Franz Josef Land and Novaya Zemlya archipelagos, located to the east and less exposed to the Atlantic Ocean, are much less studied [11,13]. In order to investigate the relationship between the soil carbon dioxide emission process and soil microbial diversity, we conducted a study of soil samples from the Novaya Zemlya and Franz Josef Land archipelagos, in which we analyzed bacterial diversity, soil parameters and soil carbon dioxide emission. 

## 2. Materials and Methods

### 2.1. Soil Properties

Soil carbon dioxide emission measurements, determination of the soil properties and sampling of microbial communities were conducted during the Arctic Floating University cruise in summer 2021. On Novaya Zemlya archipelago (Northern Island, Cape Zhelaniya) (76°56 N, 68°32 E), measurements and sampling were carried out on 17 June 2021 from 13:00 to 19:00. Measurements and sampling were conducted on Heiss Island (Franz Josef Land) (80°37 N, 58°03 E) on 20 June 2021 from 10:00 to 16:00, and on Hooker Island (Tikhaya Bay, Franz Josef Land) (80°20 N, 52°46 E) on 21 June 2021 from 10:00 to 16:00. On each of the islands, there was one monitoring plot of 5 m × 5 m with a spacing of 1 m established. The total number of sampling points at each monitoring plot was 25. Soil moisture was determined using an OHAUS MB35 Moisture Analyzer. The pH was determined in a supernatant of soil and water in a ratio of 1:2.5 by pH meter ANION 4100 (Russia). The pH of the aqueous suspension of mineral soil horizons was determined using the potentiometric method [22].

### 2.2. Carbon Dioxide Emission

Determination of carbon dioxide emission was conducted by the closed non-steady-state non-through-flow chambers method during daytime hours from the soil surface with removed vegetation cover [23,24]. For this purpose, cylindrical metal chambers were made of stainless steel. A circular cover was hermetically soldered to the cylinder on the upper side, and the cylinder was open on the lower side facing the soil. The volume of the chambers was 0.9 L, the diameter of the chamber was 100 mm, and the height was 124 mm. For gas sampling, 1 cm diameter holes were made on the upper side of the chambers and closed with butyl rubber plugs. The vegetation cover was preliminarily removed at the place of chamber installation, and the chambers were installed 1–2 cm deep into the soil. Immediately after installation and after 2 h of exposure, 10 cm^3^ gas samples were taken from the chamber with a syringe. The air in the chamber was stirred by pumping the syringe piston three times before each sampling. After that, the selected gas sample was injected into a gas analyzer, and the gas concentration in the chamber at the initial moment and at the end of the exposure was determined. When the weather was clear and cloudless, the chambers were covered with reflective material to avoid a temperature increase due to solar heating. The CO_2_ concentration was measured using an RMT DX6210 portable gas analyzer (RMT Ltd.). The CO_2_ flux from the soil into the chamber was calculated using the formula following the equation of state for an ideal gas [23].

Q = (ΔX × P × M × h)/(100 × R × T × Δt)

where Q [mgCO_2_/(m^2^ × h)] is the value of gas flux, ΔX [%] is the increase in bulk CO_2_ content, P [kPa] is barometric (atmospheric) pressure, h [cm] is the height of chamber from the soil surface, T [K] is the Kelvin temperature, Δt [h] is the time interval, R = 8.31 J/(mol × K) is the universal gas constant, and M [g/mol] = 44 is the carbon dioxide molecular weight.

### 2.3. Carbon Content in Soils

The carbon content of water-extractable organic carbon (WEOC) was determined in a 0.05 M K_2_SO_4_ solution [25,26]. A total of 5 g of fresh soil samples were mixed with 0.05 M K_2_SO_4_ solution in a 1:25 ratio, and the suspensions were shaken for 1 h on the rotator. The suspensions were then filtered through folded filters with a pore size of 3–5 μm that had been prewashed with bi-distilled water. The extracted carbon content of the extracts was determined using a TOC-VCPN automatic analyzer (Shimadzu). It should be noted that in this method, the low-concentration K_2_SO_4_ solution also serves as a coagulation agent of soil colloids, so the extracted carbon can be considered water extractable (WEOM) [27].

Determination of the microbial biomass carbon (MBC) content was carried out by the fumigation–extraction (FE) method [27,28]. In this work, fumigation of soil samples was carried out in desiccators at room temperature and natural moisture for 1 day, using the amylene-stabilised chloroform vapor without ethanol. The components of microbial cells treated by chloroform vapor were extracted with a 0.05 M K_2_SO_4_ solution and filtered, and the extracted carbon content of the extracts was determined with a TOS-VCPN automatic analyzer (Shimadzu) [27]. The MBC was calculated as C mic = Fc/kec, where Fc is the difference between the carbon content of fumigated and non-fumigated samples, and kec is a correction factor indicating incomplete carbon extraction from soils [29]. A correction factor of 0.45 kec was used [30]. In the present study, soil samples were kept at natural moisture and 4 °C storage temperature. This is because the drying–humidifying (when air-dried samples are used) and freezing–thawing (when fresh samples are stored at temperatures below 0 °C) processes result in the destruction of the microbial cell walls in the soil samples. This may lead to erroneous results in the measurement of microbial carbon. The accuracy of measurements is also affected by the storage time of soil samples. Therefore, the determination of MBC was always carried out within 1 month of sampling [31].

### 2.4. DNA Isolation, 16S Amplicon Library Preparation and Sequencing

Soil samples for DNA analysis were flash-frozen and kept at −20 °C until analysis. DNA from frozen soil samples was isolated using the DNeasy PowerSoil Pro Kit (Qiagen, Germany), according to the manufacturer’s instructions. The quality and concentration of DNA were assessed spectrophotometrically by a NanoDrop™ 8000 instrument (ThermoFischer Scientific, Waltham, MA, USA). Two nanograms of DNA were used as an input for the library preparation. The amplicon libraries of the hypervariable V4 region of the 16S rRNA gene were prepared using a two-stage PCR strategy with the primers 515F [32] and Pro-mod-805R [33]. PCR of every DNA sample was carried out in duplicate; the detailed amplification protocol is described previously [34]. The libraries were checked with agarose gel and pooled equimolarly. The final pool was purified with the Cleanup Mini PCR Purification Kit (Evrogen, Russia) according to the manufacturer’s instructions. Sequencing was performed with the MiSeq™ Personal Sequencing System (Illumina, San Diego, CA, USA) using the 156-bp paired-end reads.

### 2.5. Data Analysis

To analyze soil and environmental parameters, we used methods of descriptive statistics, comparison of mean values with Student’s parametric test (t-criterion) and Wilcoxon’s nonparametric criterion, correlation analysis including determination of Spearman’s rank correlation coefficient, forward stepwise multiple linear regression analysis and mean separation analysis using the 1-way ANOVA test. The chosen significance level was α = 0.05. Statistica 7.0 was used for statistical data processing.

Obtained sequence reads were preprocessed by removing primer sequences using Cutadapt software [35]. Read pairs lacking at least one of the primer sequences were excluded from further analysis. Remaining read pairs, corresponding to target amplicon, were demultiplexed by deML [36]. 

Analyses of microbial community composition, alpha- and beta-diversity metrics and statistics were performed using R (version 4.2.0). Primary data analysis, including filtering, merging, chimera removal and generation of amplified sequence variants (ASV) was carried out using DADA2, according to the published workflow [37,38,39]. Taxonomy assignment of generated ASVs was provided using the Silva138 16S rRNA database [40]. In cases where the taxonomy was defined ambiguously, an additional taxonomy check was performed using the NCBI Blast against nr database. In silico decontamination was performed with the decontam library [41]. Rarefaction, diversity analysis and visualization of microbial community composition were performed with microeco, vegan and pheatmap R packages [41,42,43]. 

## 3. Results

### 3.1. Soil Properties

On Novaya Zemlya, soil samples were identified as oxyaquic cryosol (arenic), which consisted of a 2–4 cm thick organogenic horizon with the mineral horizon beneath. The soil cover of Franz Josef Land archipelago was represented by an alternation of reductaquic cryosol (arenic), haplic cryosol (arenic) and oxyaquic cryosol (arenic) (Heiss Island tundra and wasteland). 

Soil temperatures at a depth of 10 cm at the monitoring sites on Novaya Zemlya and Hooker Island were not statistically different (Table 1). They are characterized by low spatial variability (coefficient of variation is 5%) whose distribution type is normal. Soil temperature varied from 3.0 to 3.7 °C, with an average of 3.3 ± 0.2 °C at monitoring sites on Novaya Zemlya and Hooker Island. Soils at the monitoring site on Heiss Island at 10 cm depth are slightly warmer (4.6 ± 0.3 °C), and are characterized by a greater thickness of the seasonally thawed layer. 

The soils on the Heiss and Hooker Islands were characterized by a near neutral pH (Table 1). The soils on Novaya Zemlya showed higher pH values, which were caused by wide distribution of carbonate marine deposits along the shores of the archipelago. The soils of the monitoring sites on all the studied islands differed significantly in soil moisture (Table 1). The highest soil moisture values were characteristic of the monitoring site on Hooker Island, and was likely caused by the close occurrence of permafrost and an active thawing process which is typical of late June. These indicators were characterized by low spatial variability, and the type of distribution is normal. All studied environmental factors were characterized by low spatial variability caused by the homogeneous character of soil and vegetation cover, as well as by the low thickness of the organogenic horizon of soils. 

### 3.2. Carbon Content

**Figure 1:**
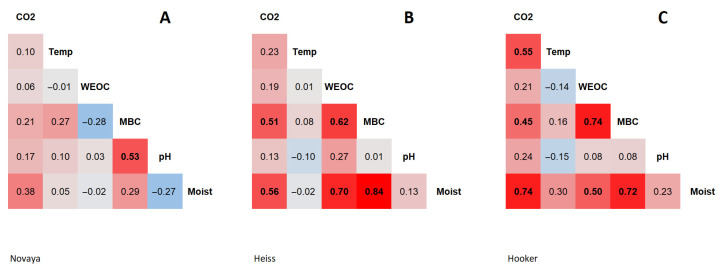
Heatmap of correlations between soil parameters: (**A**)—Novaya Zemlya, (**B**)—Heiss Island, (**C**)—Hooker Island. CO_2_—CO_2_ emission, Temp—temperature, WEOC—water-extractable organic carbon, MBC—microbial biomass carbon, Moist—moisture.

The content of WEOC in soils at the monitoring site on the Novaya Zemlya varied from 15 to 39 mg C/kg soil, and averaged 29 ± 6 mg C/kg soil (n = 25) (Table 2). WEOC content in soils at the Heiss Island ranged from 16 to 85 mg C/kg soil, and averaged 42 ± 16 mg C/kg soil (n = 25). This parameter was characterized by low spatial variability. The coefficient of variation averaged 21% on Northern Island and 38% on Heiss Island. The WEOC in soils at the Hooker Island varied widely from 22 to 210 mg C/kg soil, and averaged 60 ± 36 mg C/kg soil (n = 25). This parameter was characterized by high spatial variability, with a coefficient of variation at 60%. The distribution of extractable carbon content values was asymmetrical at all monitoring sites; the median was shifted to low values. No statistically significant relationship was found between the WEOC of soils of Novaya Zemlya and other soil properties (Figure 1, Supplementary Tables S1–S3). The spatial distribution of the WEOC in soils of the Heiss and Hooker Islands correlates with the soil moisture: Heiss Island, r = 0.70, *p*-value < 0.05; Hooker Island, r = 0.51, *p*-value < 0.05. 

The MBC in soils of the Novaya Zemlya varied from 137 to 561 mg C/kg soil, and averaged 304 ± 107 mg C/kg soil (n = 25). The MBC in soils of Heiss Island varied within a wide range from 43 to 1300 mg C/kg soil, and averaged 305 ± 387 mg C/kg soil (n = 25). The MBC of microbial biomass in soils of Hooker Island varied from 12 to 1400 mg C/kg soil, and averaged 234 ± 353 mg C/kg soil (n = 25). This parameter in the soils of the Heiss and Hooker Islands was characterized by a high spatial variability. The coefficient of variation was 127% on Heiss Island and 151% on Hooker Island. The spatial distribution of the MBC in soils of the Novaya Zemlya was determined to a greater extent by soil pH (r = 0.53, *p*-value < 0.05). On the Heiss and Hooker Islands the spatial distribution of the MBC was determined mostly by soil moisture; on Heiss Island, r = 0.84, *p*-value < 0.05, and on Hooker Island, r = 0.72, *p*-value < 0.05 (Figure 1, Supplementary Tables S1–S3). The distribution of MBC values was asymmetrical at all monitoring sites; the median was shifted towards low values. 

Analysis of data on WEOC and MBC content in soils shows their high spatial variability in all ecosystems studied. The spatial distribution of the WEOC and MBC content of soils on Heiss and Hooker Islands was found to be determined by soil moisture. This pattern is not characteristic of the soils on Novaya Zemlya, which is most likely due to the relatively low soil moisture values on this island compared to those on the Heiss and Hooker Islands (14.4%, 28.4% and 45.6%, respectively).

### 3.3. Carbon Dioxide Emission

Carbon dioxide emission by soils of Novaya Zemlya varied within a wide range from 0 to 122 mg CO_2_/m^2^ × h, and averaged 31 ± 41 mg CO_2_/m^2^ × h (n = 25). Carbon dioxide emissions on Heiss Island varied within a range of 0 to 250 mg CO_2_/m^2^ × h, and averaged 54 ± 67 mg CO_2_/m^2^ × h (n = 25). On Hooker Island, carbon dioxide emission varied from 10 to 278 mg CO_2_/m^2^ × h, and averaged 106 ± 78 mg CO_2_/m^2^ × h (n = 25). This indicator was characterized by a high spatial variability with the coefficient of variation of 134% (Novaya Zemlya), 124% (Heiss Island) and 74% (Hooker Island). The distribution of CO_2_ emission values was asymmetrical, and the median shifted towards low values. No statistically significant relationship was found between the CO_2_ emissions and measured soil parameters of Novaya Zemlya (Figure 1, Supplementary Tables S1–S3). The values and variability of this parameter are probably influenced by a wider range of environmental factors. In the soils of the Heiss Island, a CO_2_ emission correlated with the soil moisture (r = 0.56, *p*-value < 0.05) and MBC (r = 0.51, *p*-value < 0.05). In the soils of the Hooker Island, the emission correlated with soil moisture (r = 0.74, *p*-value < 0.05), temperature (r = 0.55, *p*-value < 0.05) and MBC (r = 0.45, *p*-value < 0.05). The higher CO_2_ emission values in soils on Hooker Island compared to soils on other islands may be due to the southern exposure of the slope on which the monitoring site was located, and thus to the more favorable thermal conditions for the activity of soil microbiota. 

### 3.4. Microbial Diversity Analysis Using 16S Metabarcoding

High-throughput sequencing of V4 16S rRNA gene amplicons from soil samples of Franz Josef Land and Novaya Zemlya produced 1,212,919 raw read pairs. After demultiplexing and read-filtering procedures, 13,950 read pairs were obtained, on average, for each sample. ASV rarefaction curves built with the vegan R package reached a saturation of approximately 5000 reads for all sequenced samples, indicating the sufficiency of sequencing depth [42] (Supplementary Figure S1). In total, 2114 amplicon sequence variants (ASV) were obtained for Franz Josef Land (35 samples) and 671 ASVs were obtained for Novaya Zemlya (25 samples). 

**Figure 2:**
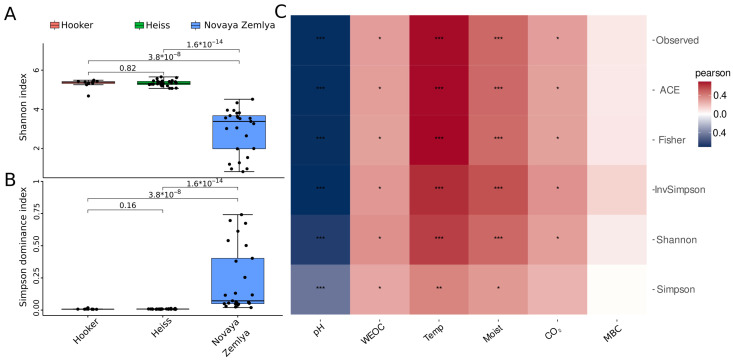
Visualization of alpha-diversity analysis of soil microbial communities of Barents Sea islands: (**A**,**B**)—boxplots of diversity metrics compared between Franz Josef Land and Novaya Zemlya islands, (**C**)—heatmap of Pearson’s correlation coefficients between alpha-diversity metrics and environmental parameters. WEOC—water-extractable organic carbon, Temp—temperature, CO_2_—carbon emission, Moist—moisture, MBC—microbial biomass carbon; *p*-values are labeled as follows: *p* < 0.05—*; *p* < 0.01—**; *p* < 0.001—***.

Analysis of alpha-diversity metrics performed by the *microeco* package showed that the level of microbial diversity in the Franz Jozef Land samples was significantly higher than that in the Novaya Zemlya samples (Figure 2, Supplementary Table S4), which might be explained by clear dominance of certain taxa in the Novaya Zemlya samples. In turn, the differences in alpha diversity metrics between the Franz Josef Land islands were insignificant. Correlation analysis between alpha diversity metrics and environmental parameters (Figure 2C) showed a high level of correlation between alpha diversity metrics, temperature and soil moisture (the Pearson’s coefficient values for the Shannon index were 0.58 and 0.46, respectively, with *p*-values < 0.001). A lower level of correlation was found between alpha diversity and CO_2_ emission (the Pearson’s coefficient value for the Shannon index was 0.29, with *p*-value < 0.05) and WEOC (the Pearson’s coefficient value for the Shannon index 0.34, *p*-value < 0.05). A strong negative correlation was discovered between alpha diversity and soil pH (the Pearson’s coefficient value for the Shannon index was −0.63, *p*-value < 0.001).

**Figure 3:**
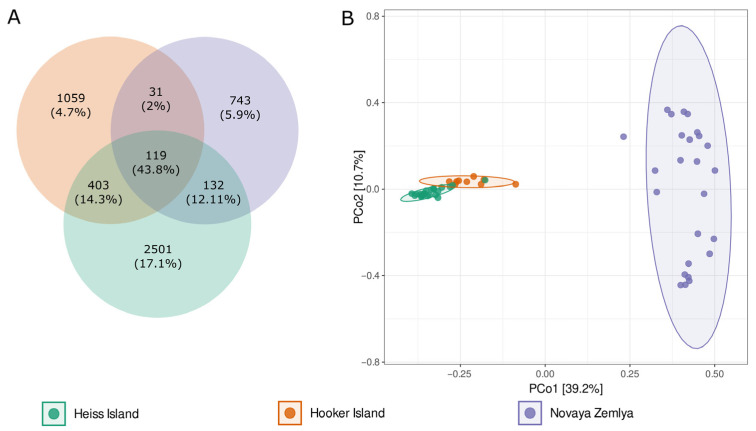
(**A**)—Venn diagram of the core, shared and island-specific ASVs for three islands involved in the study, (**B**)—Principal coordinates analysis (PCoA) ordination of variation based on weighted Bray–Curtis dissimilarity.

Analysis of the core, shared and island-specific microbiome revealed 119 ASVs common for all islands and representing 43.8% of all reads. Novaya Zemlya and Hooker Island showed a similar share of island-specific ASVs, representing 14.9 and 21.2% of all reads, respectively. Heiss Island, in turn, possessed a larger fraction of the specific microbiome (2501 ASVs, representing 50.1% of reads). The percentage of ASVs shared between Hooker and Heiss Islands was much bigger than the total share between Novaya Zemlya and other islands (8.1 vs. 3.2%), assuming that the microbial community composition of Novaya Zemlya is distinct from the communities of Franz Josef Land (Figure 3).

This observation was supported by a PERMANOVA analysis, performed by *vegan* function *adonis2*, based on the Bray–Curtis distance matrix: the R^2^ of *adonis* results was 0.45, *p*-value 0.001). Principal coordinates analysis (PCoA) ordination also showed distinct segregation of samples taken from different archipelagos (Figure 3).

### 3.5. Microbial Community Composition

**Figure 4:**
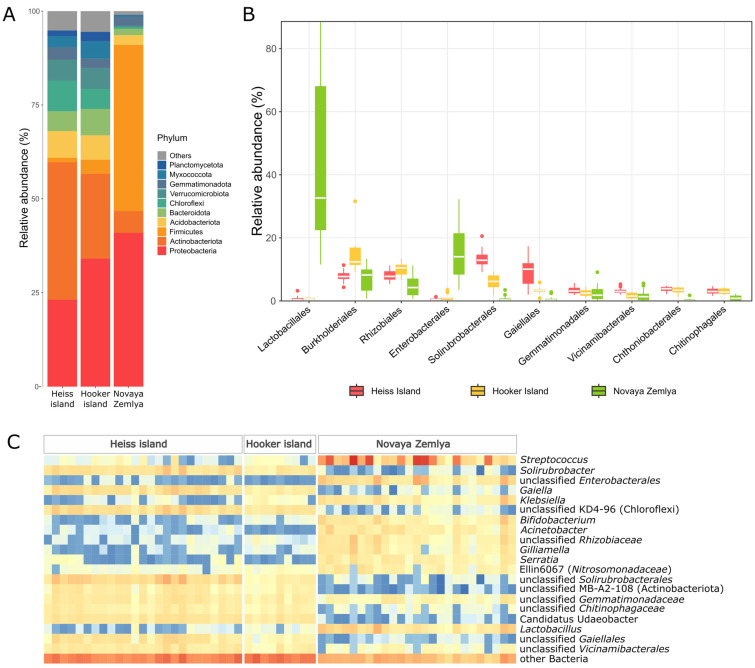
Microbial abundance in the soil samples: (**A**)—The proportion of the bacterial phyla in the soils, (**B**)—Boxplot of the top 10 order abundances in each sample, (**C**)—Abundance heatmap of the genera, comprising more than 5% at least in one of the samples.

The composition of the microbial communities differed significantly in the soil samples from the archipelagos. Novaya Zemlya samples were clearly dominated by the phylum firmicutes mainly represented by the *Lactobacillales* order (Figure 4), including highly abundant genera *Streptococcus* (up to 86.3% of all reads) and *Lactobacillus* (up to 19.2%). The second most common phylum was proteobacteria dominated by representatives of *Enterobacterales*. Additional identification using BLAST showed that this group includes members of the family *Yersiniaceae*, closely related to members of the genera *Rahnella, Serratia* and *Yersinia*, which include pathogenic bacteria as well as bacteria found in animal gastrointestinal tract, soil and aquatic ecosystems. The representatives of the *Acinetobacter* genus, often associated with a ruminant microbiome, varied between 0.01% and 11.3% [44,45]. Other gut microbiota-associated proteobacterial genera detected in Novaya Zemlya samples included *Klebsiella*, *Morganella*, *Serratia*, and *Snodgrassella.* Interestingly, ASVs, belonging to *Gilliamella* genus in which valid and cultivated representatives are isolated exclusively from bee gut [46], were found in significant amounts (1.2% on average). ASVs from the *Rhizobiaceae* family have also been associated with the intestines of bees, and additional analysis has shown a high degree of similarity with the *Bartonella apihabitans* strain isolated from the bee gut. Additionally, identified among the proteobacteria are members of the family *Nitrosomonadaceae*, all of whose cultivated representatives are lithoautotrophic ammonia oxidizers [47]. The phylum actinobacteriota in Novaya Zemlya soil samples was represented mainly by *Bifidobacterium* (maximum 9.29%). Thus, a comparison of the dominant taxa in the soils of Novaya Zemlya with the literature data shows that microorganisms frequently found in the gastrointestinal tract of animals and fecal microbiota dominate in soil samples from this island [48,49].

Heiss island samples were dominated by actinomycetota, reported to be one of the major constituents of the soil core microbiome [50]. This phylum was represented mostly by the genera *Solirubrobacter, Gaiella* and yet uncultured representatives of *Solirubrobacterales,* (up to 6.87, 10.04 and 12.2 of bacterial communities, respectively). Additionally, an uncultured class MB-A2-108, often associated with deeper layers of floodplain soils [51], was found in significant amounts (2.47% on average). Proteobacteria phylum representatives took second place in abundance from actinomycetota. The most frequently encountered taxa for gammaproteobacteria were representatives of uncultured families SC-I-84, A21b and MND1 (maximum 1.68% and 1.49%, respectively). Alphaproteobacteria were dominated by *Xanthomonadaceae* and *Rhodomicrobiaceae* families. 

The uncultured class KD4-96 of chloroflexi phylum, reported to be associated with contaminated soils [52], was also quite abundant in the communities of Heiss Island. Up to 7.52% of all reads were assigned to this class in some samples. Representatives of the phyla acidobacteriota, bacteroidota, and verrucomicrobiota were found in the soil samples of Heiss Island in insignificant amounts. Acidobacterioidota in most cases were associated with uncultured representatives of *Viciamibacterales* order (1.9–4.51% of all reads). Bacteroidota were represented by unclassified *Chitinophagaceae* (up to 2.79% of all reads) and vrrucomicrobiota by *Candidatus Udaeobacter* (4.79%). Additionally, significant amounts of reads assigned to the phyla gemmatimonadota, nitrospirota, myxococcota, and desulfobacterota were detected. Heiss Island soils were the only group of samples possessing archaeal ASVs in significant (>1%) amounts. These ASVs were associated with unidentified representatives of *Nitrososphaeraceae* family.

The set of taxa detected in the microbial communities of Hooker Island soils was generally the same as on Heiss Island; however, archaea was almost absent in all samples. On Hooker Island, as opposed to Heiss, proteobacteria dominated over actinomycetota. The most abundant proteobacterial genus was *Polaromonas* (up to 6%)*,* common for the Arctic soils [53]. In turn, *Gaiella,* which according to some reports is quite abundant in polar Antarctic soils [54], dominated among actinomycetes. Other phyla were presented approximately in the same relative quantities as in the Heiss Island community. Thus, the samples from Franz Josef Land are dominated by taxonomic groups characteristic of soil communities [55]. 

## 4. Discussion

As a result of this study, it was found that the highest levels of carbon dioxide emissions and microbial diversity were observed on the Franz Josef Land islands, while the lowest values were observed on Novaya Zemlya. This suggests a possible relationship between biodiversity and soil ecosystem functioning, as has been shown previously for soils under different climatic conditions [56,57,58].

In general, the soils of the islands studied are characterized by high heterogeneity, largely similar physico-chemical characteristics, low extractable and microbial carbon content and a low thickness of the organogenic layer typical of the high Arctic [59]. The data we obtained from our studies on the content of microbial carbon are comparable to the Arctic desert and tundra ecosystems, according to the literature data [60,61,62]. 

The obtained CO_2_ emission values generally correspond to the level of carbon dioxide emission from the soils of the Arctic and subarctic zones [63,64,65,66,67]. The low values of CO_2_ emission are mainly due to the specific features of the study area: permafrost close to the soil surface, low air temperatures and a high moisture content in the soil strata. In June, when the sampling campaign was performed, only parts of the Arctic islands are free of snow, and the thickness of the seasonally thawed layer in the soil profile is not significant. The peak of the vegetation season at the study sites is in July, and in this period, an increase in CO_2_ fluxes from soil can presumably be observed. Considering that CO_2_ emissions were measured before the peak of the vegetation season, it can be assumed that the studied ecosystems could be characterized by a more significant carbon flux into the atmosphere. 

Comparison of soil CO_2_ emission, WEOC and MBC with environmental parameters showed that the most significant level of correlation of these parameters was observed in the soil moisture on Franz Josef Land. MBC and temperature were the next most important factors influencing soil CO_2_ emission on Franz Josef Land. In the case of Novaya Zemlya, no significant correlation between these parameters and environmental parameters was found. This may be explained by the lower values of average soil moisture on Novaya Zemlya compared to Franz Josef Land. 

The only significant factor influencing MBC content on Novaya Zemlya is soil pH; no significant influence was detected for other parameters. pH is considered one of the most important determinants of soil microbiota composition [68,69]. Previous studies have shown that in Arctic soils, pH is a more significant factor influencing the composition of soil microbial communities, while total organic carbon, moisture and electrical conductivity have less influence [70]. The specific mechanisms of pH influence on the composition of soil microbial communities are not currently known. Two main hypotheses are usually considered. According to the first one, pH directly affects the survival of cells with narrow pH tolerances [71]. According to the second hypothesis, pH affects the solubility, concentration and availability of chemical compounds, particularly calcium, phosphorus, magnesium, potassium and other elements, which in turn affects the activity and survival of microbial cells [68].

Comparison of microbial community composition and soil parameters showed a significant positive correlation with soil moisture and temperature and a significant negative correlation with pH. A comparison of the taxonomic composition of the soils of the studied islands with earlier data has shown that the soils of Franz Josef Land are dominated by representatives of microbial groups that are characteristic of Arctic soil communities. These are proteobacteria, actinomycetota, firmicutes and bacteroidetes [72]. Previous studies on the physiology of cultured microorganisms isolated from cryogenic soils of Novaya Zemlya have shown that they are tolerant to a wide range of factors, including wide temperature and pH ranges, at which cultures grow moderate halotolerance properties and multiple antibiotic resistance [72]. 

In contrast to Franz Josef Land, the samples from Novaya Zemlya showed a predominance of taxa characteristic of the digestive tract of animals. These include groups such as *Streptococcus*, *Lactobacillus* and others. A possible explanation for this observation may be the different faunal composition between the archipelagos. In particular, the northern tip of Novaya Zemlya is within the reindeer range, whereas, there is currently no reindeer population on Franz Josef Land [73,74]. The reindeer is the largest and often the only large herbivore in many areas of the Arctic, and has a significant influence on the structure of plant communities and ecosystem processes [75]. Radiocarbon dating of reindeer remains found on Franz Josef Land showed that they were present on the archipelago between 6400 and 1300 years ago, when climatic conditions on Franz Josef Land were milder. They disappeared from the archipelago about 1000 years ago (c. 1000 cal. BP) during the onset of the neoglacial period [76]. However, on other Arctic archipelagos (Svalbard, Novaya Zemlya, Novosibirsk Islands, Severnaya Zemlya, Wrangel Island, etc.) reindeer populations have survived to the present day. The presence of reindeer may have complex effects on soil microbiota composition through trampling and eating vegetation, and the inputs of biogenic elements and microorganisms from the gastrointestinal tract [77,78].

## 5. Conclusions

The results of studies of diversity and carbon dioxide emission by microbial communities in the archipelagos of Novaya Zemlya and Franz Josef Land show a possible relationship between CO_2_ emission and diversity indicators. The highest diversity and CO_2_ emission levels were observed on the Hooker and Heiss Islands of the Franz Josef Land archipelago, while the diversity and CO_2_ emission levels were lower on Novaya Zemlya. At the same time, the sampling site on Novaya Zemlya is more than 400 km south of the studied islands of the Franz Josef Land archipelago. Soil moisture and temperature were the main parameters influencing the composition of soil microbial communities on both archipelagos. Additionally, soil moisture was the main factor influencing carbon emission on Franz Josef Land, whereas in the drier soils of Novaya Zemlya, the role of moisture was less prominent. The data obtained show that higher CO_2_ emission levels and community diversity on the studied islands are influenced mostly by a number of local factors such as soil moisture, microclimatic conditions, different patterns of vegetation and fecal input from animals such as reindeer.

## Figures and Tables

**Table 1:** Soil properties of the study areas.

Island	Soil Temperature (10 cm Depth), °C	Soil pH	Soil Moisture, %	Active Layer Thickness, cm	Organic Layer Thickness, cm
Novaya Zemlya	3.3 ± 0.2 ^1^	8.6 ± 2.6	14.4 ± 1.3	30 ± 5	3.0 ± 1.3
Heiss Island	4.6 ± 0.3	7.3 ± 0.1	28.4 ± 3.3	35 ± 5	3.6 ± 1.3
Hooker Island	3.3 ± 0.2	6.9 ± 0.1	54.6 ± 5.4	15 ± 5	2.3 ± 0.9

^1^ Mean ± standard deviation.

**Table 2:** Mean values of the CO_2_ emission, WEOC and MBC in soil samples.

Island	CO_2_ Emission, mg CO_2_/m^2^ × h	WEOC, mg C/kg Soil	MBC, mg C/kg Soil
Novaya Zemlya	31 ± 41 ^1^	29 ± 6	304 ± 107
Heiss Island	54 ± 67	42 ± 16	305 ± 387
Hooker Island	106 ± 78	60 ± 36	234 ± 353

^1^ Mean ± standard deviation.

## Data Availability

FASTQ sequences of this metagenomic sample have been deposited in the NCBI Short Read Archive under BioProject PRJNA 913966.

## Supplementary Materials

The following supporting information can be downloaded at: https://www.mdpi.com/article/10.3390/microorganisms11020482/s1, Figure S1: Rarefaction curves of the studied samples; Table S1: Novaya Zemlya; Table S2: Heiss Island; Table S3: Hooker Island; Table S4: Microbial diversity indices.
