# Supplementary material for: Carbon Emission and Biodiversity of Arctic Soil Microbial Communities of the Novaya Zemlya and Franz Josef Land Archipelagos
Source: Microorganisms. 2023 Feb 15;11(2):482. doi: 10.3390/microorganisms11020482 (PMC9962458; doi:10.3390/microorganisms11020482)
Supplement: Supplementary file 1 [file microorganisms-11-00482-s001.zip › Suppl Tables S1-S4.pdf]

Supplementary Table S1. Novaya Zemlya

	CO2 emission	Temperature	WEOC	MBC	pH	Moisture
**CO2 emission**	1.000000					
**Temperature**	0.096006	1.000000				
**WEOC**	0.064681	-0.006803	1.000000			
**MBC**	0.210795	0.269766	-0.277711	1.000000		
**pH**	0.171284	0.104237	0.034641	**0.534143**	1.000000	
**Moisture**	0.376780	0.049045	-0.016098	0.293271	-0.274802	1.000000

Supplementary Table S2. Heiss Island

	CO2 emission	Temperature	WEOC	MBC	pH	Moisture
**CO2 emission**	1.000000					
**Temperature**	0.230802	1.000000				
**WEOC**	0.185400	0.008384	1.000000			
**MBC**	**0.512794**	0.075314	**0.621024**	1.000000		
**pH**	0.128127	-0.102226	0.272073	0.014930	1.000000	
**Moisture**	**0.558109**	-0.021042	**0.702022**	**0.840699**	0.126845	1.000000

Supplementary Table S3. Hooker Island

	CO2 emission	Temperature	WEOC	MBC	pH	Moisture
**CO2 emission**	1.000000					
**Temperature**	**0.548832**	1.000000				
**WEOC**	0.212303	-0.141420	1.000000			
**MBC**	**0.446942**	0.157067	**0.744983**	1.000000		
**pH**	0.242071	-0.146482	0.077014	0.082068	1.000000	
**Moisture**	**0.737586**	0.301643	**0.502596**	**0.716041**	0.227703	1.000000

Supplementary Table S4. Microbial diversity indices

	Observed	Chao1	ACE	Shannon	Simpson	InvSimpson	Fisher
**Hooker_Island_1**	420.00000	423.00000	420.91804	5.55546	0.99281	139.02334	87.22105
**Hooker_Island_2**	275.00000	276.50000	275.64453	4.90819	0.98335	60.05830	54.68733
**Hooker_Island_3**	478.00000	480.50000	478.72967	5.76626	0.99523	209.44805	98.00051
**Hooker_Island_4**	468.00000	471.00000	468.92422	5.66257	0.99392	164.60530	93.74004
**Hooker_Island_5**	535.00000	535.54545	535.65185	5.85494	0.99534	214.62439	110.00272
**Hooker_Island_6**	350.00000	351.20000	350.62032	5.53143	0.99437	177.58597	74.96858
**Hooker_Island_7**	460.00000	465.25000	461.18633	5.68298	0.99440	178.64065	91.43908
**Hooker_Island_8**	464.00000	469.60000	465.33062	5.74030	0.99507	202.64778	97.23268
**Hooker_Island_9**	468.00000	473.25000	469.20480	5.72237	0.99446	180.60450	98.91427
**Novaya_Zemlya_1**	104.00000	104.00000	104.17365	2.56843	0.77284	4.40221	15.23095
**Novaya_Zemlya_10**	54.00000	54.00000	54.24541	0.89675	0.27228	1.37416	7.05127
**Novaya_Zemlya_11**	105.00000	105.00000	105.19431	3.28576	0.91735	12.09854	16.06541
**Novaya_Zemlya_12**	125.00000	125.00000	125.14798	3.13755	0.88023	8.34957	18.84468
**Novaya_Zemlya_13**	37.00000	39.00000	39.62199	0.39499	0.11286	1.12722	4.42383
**Novaya_Zemlya_14**	124.00000	125.50000	124.66860	2.79194	0.76270	4.21402	20.42766
**Novaya_Zemlya_15**	47.00000	47.00000	47.00000	0.49417	0.13718	1.15899	5.86791
**Novaya_Zemlya_16**	92.00000	92.00000	92.00000	3.17568	0.92718	13.73321	12.95456
**Novaya_Zemlya_17**	128.00000	128.00000	128.15315	3.58163	0.93574	15.56256	21.25083
**Novaya_Zemlya_18**	154.00000	154.00000	154.00000	3.77482	0.92814	13.91646	28.38889
**Novaya_Zemlya_19**	125.00000	126.00000	125.31677	1.79696	0.49591	1.98376	19.61448
**Novaya_Zemlya_2**	165.00000	165.25000	165.31517	3.15549	0.88416	8.63274	26.26483
**Novaya_Zemlya_20**	51.00000	52.00000	51.40821	0.55753	0.16723	1.20081	6.61265
**Novaya_Zemlya_21**	52.00000	53.00000	52.46918	0.49334	0.14197	1.16546	6.70702
**Novaya_Zemlya_22**	90.00000	90.00000	90.21615	1.12103	0.31135	1.45211	12.67677
**Novaya_Zemlya_23**	119.00000	119.00000	119.14626	3.43770	0.88269	8.52430	21.88189
**Novaya_Zemlya_24**	253.00000	254.00000	253.50031	4.72662	0.98039	50.99612	49.78833
**Novaya_Zemlya_25**	82.00000	82.25000	82.59439	1.24264	0.36552	1.57610	11.16341
**Novaya_Zemlya_3**	163.00000	164.00000	163.29630	3.32098	0.87502	8.00129	26.87810
**Novaya_Zemlya_4**	194.00000	194.00000	194.14925	3.34251	0.84822	6.58834	33.79352

**Novaya_Zemlya_5**	220.00000	220.00000	220.00000	4.37430	0.97411	38.62463	39.50228
**Novaya_Zemlya_6**	83.00000	83.00000	83.00000	0.70253	0.18962	1.23398	10.97295
**Novaya_Zemlya_7**	172.00000	173.50000	172.50230	3.36724	0.89511	9.53408	28.02837
**Novaya_Zemlya_8**	113.00000	113.00000	113.17979	3.52268	0.94448	18.01297	18.00565
**Novaya_Zemlya_9**	148.00000	148.25000	148.33557	3.77522	0.95910	24.45075	23.92705
**Heiss_Island_1**	489.00000	498.00000	490.78902	5.81711	0.99541	217.73495	102.41070
**Heiss_Island_10**	481.00000	494.75000	483.32777	5.67690	0.99412	170.15610	97.96096
**Heiss_Island_11**	426.00000	428.50000	426.75552	5.57507	0.99332	149.77397	86.17301
**Heiss_Island_12**	447.00000	448.87500	447.96925	5.61622	0.99367	158.07314	89.16769
**Heiss_Island_13**	430.00000	431.42857	430.77930	5.59947	0.99391	164.22225	87.28989
**Heiss_Island_14**	625.00000	638.12500	628.12891	5.93051	0.99512	204.73629	125.77947
**Heiss_Island_15**	542.00000	542.76923	542.95068	5.71526	0.99343	152.20838	106.48680
**Heiss_Island_16**	562.00000	575.20000	564.01115	5.78458	0.99416	171.11251	111.04079
**Heiss_Island_17**	602.00000	615.00000	604.98890	5.82058	0.99429	175.08519	118.07336
**Heiss_Island_18**	574.00000	579.07692	576.56150	5.75678	0.99406	168.31778	112.18740
**Heiss_Island_19**	564.00000	575.00000	566.49288	5.76393	0.99379	160.96272	111.03534
**Heiss_Island_2**	443.00000	454.25000	444.81101	5.74617	0.99534	214.77726	93.21146
**Heiss_Island_20**	529.00000	532.60000	530.66559	5.67034	0.99296	141.99055	103.93549
**Heiss_Island_21**	576.00000	577.75000	577.34477	5.85176	0.99464	186.73609	116.46463
**Heiss_Island_22**	464.00000	471.00000	465.32344	5.65035	0.99421	172.65354	93.17111
**Heiss_Island_23**	401.00000	405.20000	402.30398	5.42398	0.99185	122.77064	78.32529
**Heiss_Island_24**	429.00000	431.14286	430.08378	5.28230	0.98573	70.05607	82.52687
**Heiss_Island_25**	494.00000	509.60000	497.06622	5.67450	0.99365	157.42295	98.79735
**Heiss_Island_3**	472.00000	478.60000	474.68043	5.63902	0.99327	148.65332	96.07013
**Heiss_Island_4**	448.00000	463.00000	449.95084	5.39518	0.98814	84.29943	87.50485
**Heiss_Island_5**	364.00000	371.00000	365.09829	5.39702	0.99136	115.75816	75.02330
**Heiss_Island_6**	497.00000	502.62500	498.94537	5.85330	0.99581	238.52813	105.28619
**Heiss_Island_7**	472.00000	481.16667	474.24138	5.72508	0.99474	190.20757	99.54676
**Heiss_Island_8**	492.00000	505.00000	495.23057	5.78720	0.99507	202.67504	103.63317
**Heiss_Island_9**	579.00000	586.20000	580.44878	5.97419	0.99597	248.07042	121.54835
