# Supplementary figures and images for: Carbon Emission and Biodiversity of Arctic Soil Microbial Communities of the Novaya Zemlya and Franz Josef Land Archipelagos
Source: Microorganisms. 2023 Feb 15;11(2):482. doi: 10.3390/microorganisms11020482 (PMC9962458; doi:10.3390/microorganisms11020482)

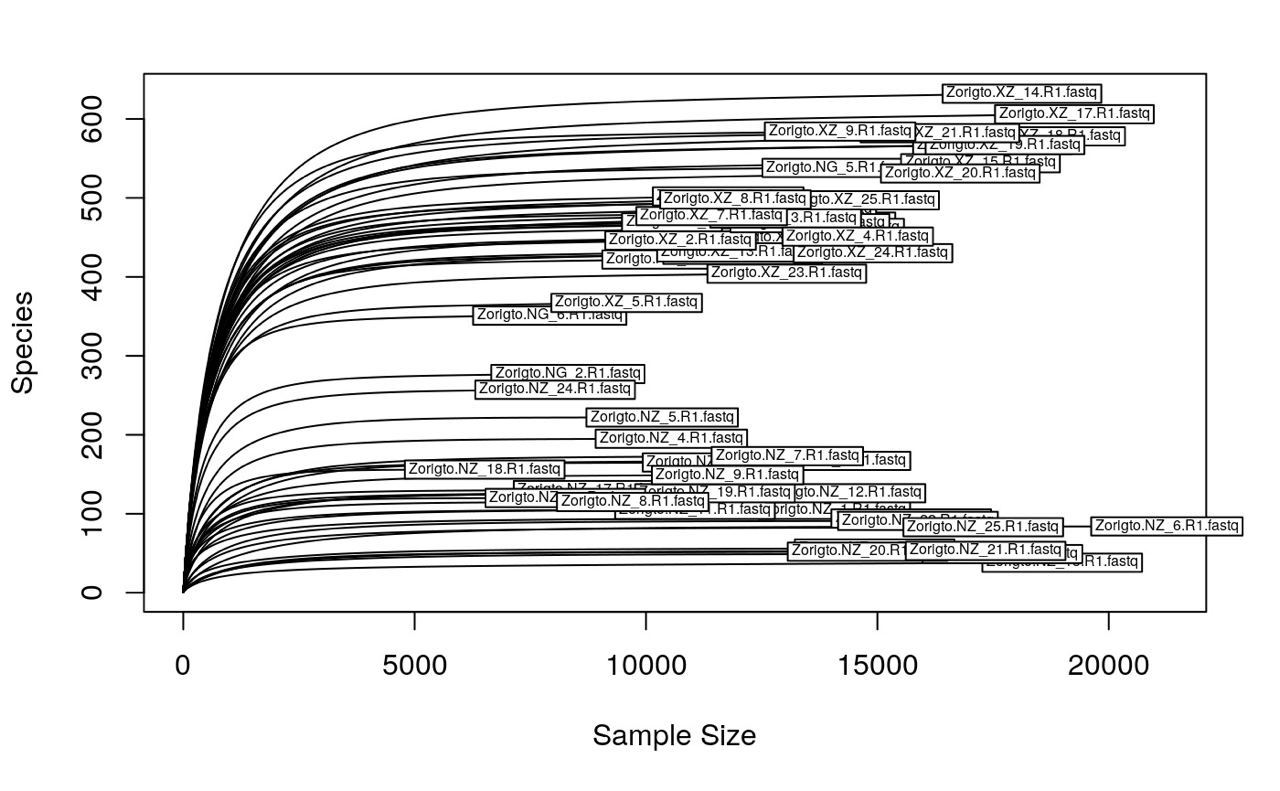

Supplement: Supplementary file 1 [file microorganisms-11-00482-s001.zip › Suppl Figure S1.jpg]
